# Directional gene flow and ecological separation in *Yersinia enterocolitica*

**DOI:** 10.1099/mgen.0.000030

**Published:** 2015-09-29

**Authors:** Sandra Reuter, Jukka Corander, Mark de Been, Simon Harris, Lu Cheng, Miquette Hall, Nicholas R. Thomson, Alan McNally

**Affiliations:** ^1^​ Pathogen Genomics, Wellcome Trust Sanger Institute, Hinxton, Cambridge CB10 1SA, UK; ^2^​ Pathogen Research Group, Nottingham Trent University, Nottingham NG11 8NS, UK; ^3^​ Department of Mathematics and Statistics, University of Helsinki, Helsinki, Finland; ^4^​ Department of Medical Microbiology, University Medical Centre Utrecht, Utrecht, Netherlands; ^5^​ Department of Infectious and Tropical Diseases, London School of Hygiene and Tropical Medicine, London WC1E 7HT, UK

**Keywords:** *Yersinia*, recombination, ecology, accessory genome

## Abstract

*Yersinia enterocolitica* is a common cause of food-borne gastroenteritis worldwide. Recent work defining the phylogeny of the genus *Yersinia* subdivided *Y. enterocolitica* into six distinct phylogroups. Here, we provide detailed analyses of the evolutionary processes leading to the emergence of these phylogroups. The dominant phylogroups isolated from human infections, PG3–5, show very little diversity at the sequence level, but do present marked patterns of gain and loss of functions, including those involved in pathogenicity and metabolism, including the acquisition of phylogroup-specific O-antigen loci. We tracked gene flow across the species in the core and accessory genome, and show that the non-pathogenic PG1 strains act as a reservoir for diversity, frequently acting as donors in recombination events. Analysis of the core and accessory genome also suggested that the different *Y. enterocolitica* phylogroups may be ecologically separated, in contrast to the long-held belief of common shared ecological niches across the *Y. enterocolitica* species.

## Data Summary

1. All raw sequence data for the genomes analysed in this publication have been previously published (Reuter *et al.*, 2014) with individual accession numbers for FastQ files and annotated genome assemblies are available in Table S1.
2. *De novo* assemblies for *Yersinia enterocolitica* genomes are available at http://pubmlst.org/yersinia/ and ftp://ftp.sanger.ac.uk/pub/pathogens/Yersinia/assemblies.
3. The Newick file for our phylogenetic tree complete with labelled strain names is also available at ftp://ftp.sanger.ac.uk/pub/pathogens/Yersinia/assemblies, and a fully interactive version of the phylogenetic tree and metadata can be found at http://microreact.org/project/EJv0OVQd.
4. Table S2 containing pangenome analysis raw data is available through Figshare http://sgm.figshare.com/articles/Alan_McNally_TableS2_xlsx/1482060.

## Impact Statement

We present data on population genomic analysis of *Yersinia enterocolitica*, an important intestinal pathogen of humans, and for decades a model organism in the study of microbial pathogenesis and evolution of mammalian virulence. By analysing the patterns of recombination in both the core and accessory genome of the species, we highlight that genetic flow in the species is directional with one phylogroup acting primarily as a genetic reservoir for the rest of the species. In addition, our detected patterns of gene flow call into question our accepted knowledge of the ecology of the species. Our data have important implications for our understanding of how recombination shapes sublineages of bacterial species and also provide a stepping-stone for investigating the ecology of bacterial subtypes through genomic data.

## Introduction

*Yersinia enterocolitica* is a Gram-negative zoonotic pathogen and an important cause of bacterial gastroenteritis in Europe (Valentin-Weigand *et al.*, 2014). The organism is ubiquitous in the environment and is commonly isolated from the tonsil tissue or intestinal tract of livestock (Fredriksson-Ahomaa *et al.*, 2000; McNally *et al.*, 2004), with most human infections epidemiologically associated with consumption of undercooked pork products (Valentin-Weigand *et al.*, 2014). Infection in humans leads to watery diarrhoea that is usually self-limiting, although post-infection sequelae such as reactive arthritis and lymphadenitis have been reported (Bottone, 1999).

**Fig. 1.**
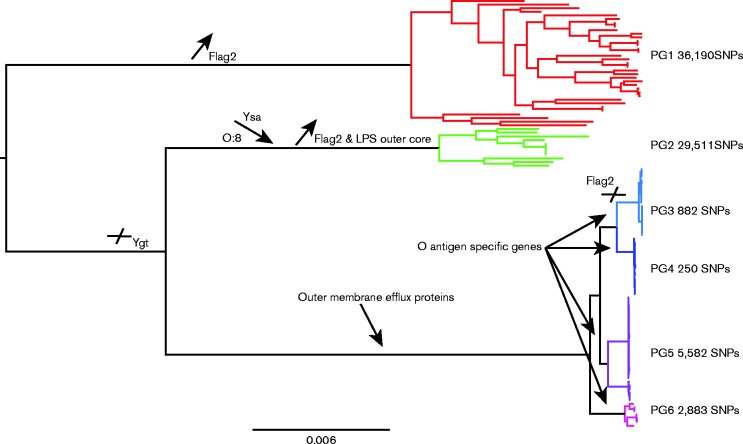
Maximum-likelihood phylogenetic tree based on a core genome alignment of 117 *Y. enterocolitica* genomes. Tips of the tree are colour coded by phylogroup, which are indicated to the right of the tree as PG1, etc. The maximum level of SNP diversity observed across each phylogroup is also denoted. The key acquisition and loss events occurring in the formation of each phylogroup as determined by accessory genome analysis are also indicated on the appropriate tree branches.

Classically, the organism has been categorized into pathovars on the basis of pathogenicity in a mouse infection model, and for routine surveillance is biotyped using a limited range of biochemical tests and serotyped. Biotype BT1B is considered to be highly pathogenic, BT1A non-pathogenic and BT2–5 low-pathogenic (Bottone, 1999). However, these descriptors have been superseded because they lack the resolution required to de-convolute the evolutionary processes that define this pathogenic species. Recent work looking at a global collection of *Y. enterocolitica* using genome sequencing covering all the major biotypes and serotypes known within the species (Reuter *et al.*, 2014) divided the population into six distinct phylogroups, as determined by the baps (Bayesian Analysis of Population Structure) program. Phylogroup PG1 contained BT1A strains and PG2 contained BT1B strains. The remaining phylogroups (PG3–6) encompass what were previously defined as the low-pathogenic BT2–5 strains (Fig. 1) and separate on the basis of serotype. This analysis showed that the pathogenic lineages evolved from a non-pathogenic ancestor. Furthermore, the descent from non-pathogenic host-generalist to pathogenic host-restricted *Y. enterocolitica* was marked by a concomitant reduction in metabolic capacity through deletion and pseudogene formation and the expansion of the IS*1667* element (Reuter *et al.*, 2014). Such a phenomenon has often been associated with enteric pathogens that have specialized to niche or host (Chain *et al.*, 2004; Cole *et al.*, 2001; Parkhill *et al.*, 2001).

In our previous analysis it was not possible to understand in any detail the events that mark the evolution of *Y. enterocolitica*. Considering its importance as a zoonotic pathogen and source of food-borne disease, we have focussed on analysing the fine-scale evolutionary flux evidenced from the genomes. Here, we show lineage-specific acquisition and loss of accessory virulence-associated genes as well as metabolic loci. We also show LPS and O-antigen operon structure concordant with the serotype-related phylogenetic structure of PG3–6. Analysis of recombination patterns, both in the core and accessory genome, suggests that the *Y. enterocolitica* phylogroups may well be ecologically separate with little detectable core genome recombination occurring between the low-pathogenic phylogroups and the non-pathogenic and high-pathogenic phylogroups. This is mirrored in the sharing of accessory genetic elements, with the majority of genetic exchange between or within phylogroups appearing on basal nodes of the tree, suggesting that what we see in extant strains is the early fixation of determinants that now define the lineages.

## Methods

### Isolates and genome sequences

All raw sequence data utilized in this study were produced as part of a previous study with full details published (Reuter *et al.*, 2014) (Table S1 and S2, available in the online Supplementary Material). *De novo* assemblies of each genome (dataset 1) were performed using Velvet and VelvetOptimizer (Zerbino & Birney, 2008). Scaffolding was done using SSPace and gaps were improved with GapFiller. Annotation was performed using Prokka (Seemann, 2014). Mapping was performed against the reference genome YE8081 using smalt (Reuter *et al.*, 2014). The phylogenetic tree (dataset 2) was constructed from a core genome alignment constructed using a Mugsy-based pipeline (McNally *et al.*, 2013a; Sahl *et al.*, 2011), with the tree inferred using the GTR-GAMMA model in RAxML version 7.2.8 (Stamatakis *et al.*, 2005).

### Pangenome analysis

Pangenome analysis (dataset 3) was performed using a pipeline developed at the Sanger Institute (Page *et al.*, 2015; https://github.com/sanger-pathogens/Bio-PanGenome). Genes shared in specific groups were filtered and are listed in separate sheets (Table S2). The number of unique and core genes was calculated by randomly adding isolates 100 times and plotting the results in r.

### Detection of recombination and direction of genetic flow

Recombination in the core genome alignment was detected using BratNextGen. A total of 20 iterations of hidden Markov model parameter estimation were performed to identify recombinations for each of PG1, 2 and 3–6. Significant (*P* ≤ 5 %) recombinations in the core genome were determined with 100 parallel permutation runs executed on a cluster computer. The negligible changes in hidden Markov model parameter values observed after ∼50 % of the iterations indicated sufficient convergence in the estimation procedure.

The direction of gene flow events was inferred as described previously (de Been *et al.*, 2013). Genome assemblies were used to create pangenomes using ls-bsr (Sahl *et al.*, 2014). The resulting pangenome matrix was used to visualize the presence or absence of all identified genetic loci in each individual genome by generation of a heatmap plot in the r package ggplot2. Shared loci of interest were identified by blast against a database of the reference *Y. enterocolitica* genomes (Reuter *et al.*, 2014). Sequences of shared loci were extracted from all assembled genomes and aligned using muscle version 3.8.31 (Edgar, 2004). Phylogenetic trees were reconstructed from the alignments using RAxML under the GTRCAT model and the trees were midpoint rooted using the Phangorn package (http://cran.r-project.org/web/packages/phangorn/index.html) in r. To determine the levels of admixture between clades, we inspected the two branches that followed the first major split closest to the root of the tree. Based on a majority voting, the branches were assigned to PG1, 2 or 3–6. Levels of admixture were calculated by dividing the number of extraneous strains in the branch by the total number of strains in the branch (extraneous+native).

## Results

### Defining diversity across the species

Our previous data reported the genomes of 117 globally and temporally dispersed *Y. enterocolitica* isolates. The phylogenetic reconstruction generated from these data showed that the pathogenic lineages evolved from a non-pathogenic ancestor. To understand in detail the variation that marks the evolution of this species, we created a pairwise distance matrix of each distinct phylogroup to quantify the varying levels of diversity, by mapping the sequence reads of these 117 isolate genomes onto the reference genome YE8081 (Table 1, Fig. 1). Our data show a maximum pairwise SNP distance in PG1 of 31 690 SNPs and in PG2 of 29 511 SNPs. Examination of the low-pathogenic groups showed that PG5 contains most diversity with a maximum SNP distance of 5582 SNPs compared with just 250 SNPs in PG4 and 882 in PG3, suggestive of a recent emergence or an evolutionary bottleneck. Attempts to date the emergence of the individual *Y. enterocolitica* lineages using beast were unsuccessful, with no resolvable temporal signature in our data.

**Table 1.**
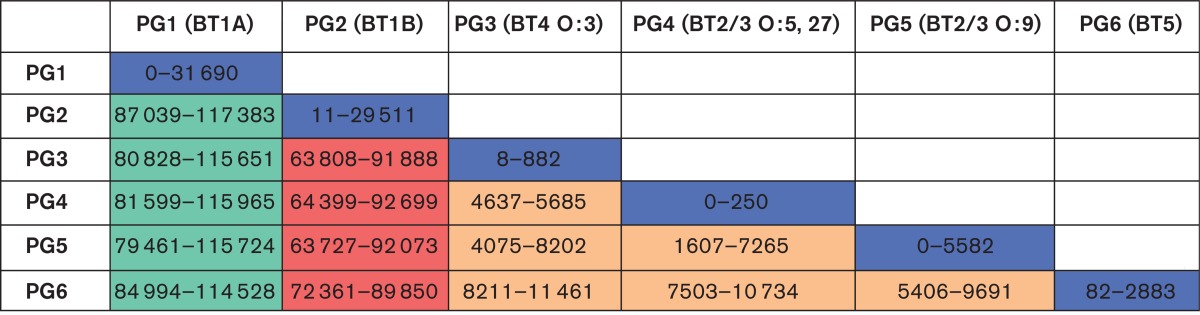
Enumeration of levels of SNP difference within and between phylogroups as determined by mapping against the reference genome YE8081 Within-phylogroup distances are highlighted in blue, non-pathogenic to pathogenic phylogroup distances are highlighted in green, high- to low-pathogenic phylogroup distances are highlighted in red and within-low-pathogenic-phylogroup distances are highlighted in orange.

### Pangenome analysis of *Y. enterocolitica* and lineage-defining regions of difference

**Fig. 2.**
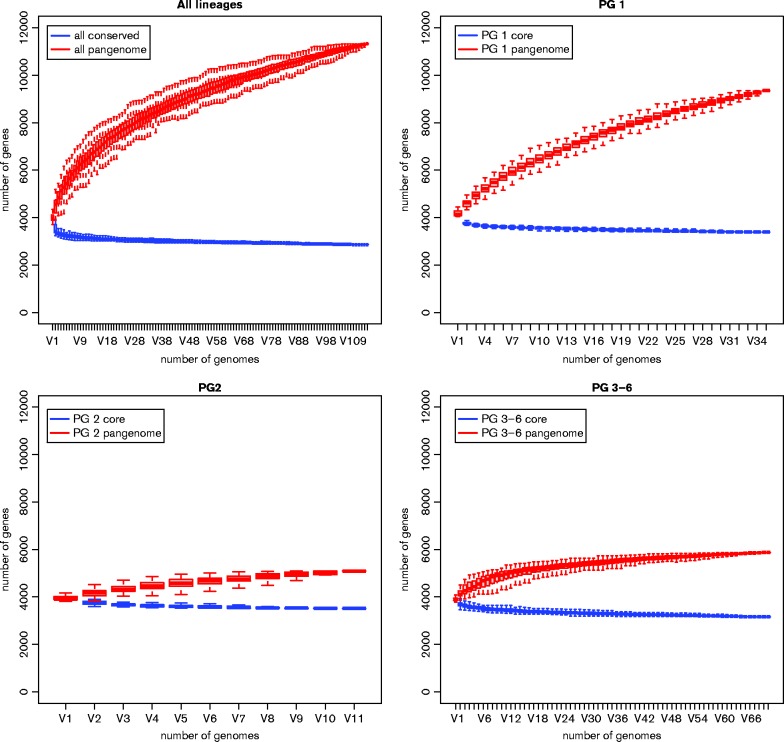
Core and pangenome statistics for all the phylogroups combined, as well as for PG1, 2 and 3–6 combined. The graphs plot number of genomes on the *x*-axis against number of genes on the *y*-axis.

Given the differing levels of genetic diversity across the phylogeny, we explored genetic distinctions between the *Y. enterocolitica* phylogroups by creating a pangenome of the species. The core and pangenomes for each of PG1, 2 and 3–6 were also determined (Fig. 2). Overall, the *Y. enterocolitica* pangenome was composed of 11 460 genes, 3181 of which were represented at least once in all strains. The pangenome for the species is intermediately open with a discovery rate of 21.92 genes per additional genome.

However, the discovery rate was different for each of the main *Y. enterocolitica* phylogroups. All of the PG1 isolates shared 3360 CDSs, making the core genome of this lineage ∼179 CDSs larger than the species core genome. Specific to the non-pathogenic PG1 strains were genes involved in metabolism, such as ABC transporters, and genes encoding arabitol and xylulose/fucose utilization as well as anaerobic nitric oxide metabolism, most likely contributing to the generalist lifestyle of this phylogroup. Furthermore, there were unique fimbriae, phages and haemolysin. The pangenome for this lineage is open (Fig. 2), exemplified by a gene discovery rate of 59.33 per genome, almost threefold greater than that for the species as a whole. The accessory genome of PG2 strains shows a similar level of diversity to PG1, with a gene discovery rate of 40.45 per genome. Genes that were specific to the high-pathogenic PG2 isolates related to its well-known virulence factors yersiniabactin encoded in the high-pathogenicity island and the Ysa type III secretion system (T3SS), as well as fimbriae and a haemolysin.

Analysis of the core and accessory genomes for PG3–6 showed evidence of very few gene gain/loss events in this lineage, consistent with the low SNP diversity and with a gene discovery rate of just 2.52 per genome in PG3. Genes specific for the low-pathogenic PG3–6 strains included gene clusters relating to haem transport (haemophore), metabolism and storage, colicin and its immunity protein, fimbriae, the insect toxin complex pathogenicity island, and the flagella cluster Flag-2, although this was partially deleted in PG3 genomes. The presence of remnant Flag-2 sequences in both PG1 and 2 suggested that Flag-2 was an ancestral element in *Y. enterocolitica* that was independently lost by these lineages.

The LPS biosynthesis genes are also phylogroup-specific, acting as defining markers of their lineage (Bengoechea *et al.*, 2004; Pinta *et al.*, 2010). *Y. enterocolitica* contains LPS outer core genes that are found in the same region between genes YE3069 and YE3088 (with respect to high-pathogenic reference genome YE8081) in all *Y. enterocolitica* phylogroups, suggesting that this is the ancestral LPS operon state. The one exception to this is in PG2, where the outer core genes have been replaced with the serotype O : 8-encoding genes, similar to the LPS structure in *Yersinia pestis*, identifying this as a likely key event in their emergence. The LPS operons found within PG3–6 phenotypically encode the O : 2a, 2b, 3, O : 5, 27, O : 3 and O : 9 O-antigen clusters. They are all located in identical positions between YE2772 and YE2779, but carry serotype-specific gene rearrangements and/or nonsense mutations that correlate with the altered serotype phenotypically displayed.

### Analysis of phylogroup-shared regions identifies key gene loss events

**Fig. 3.**
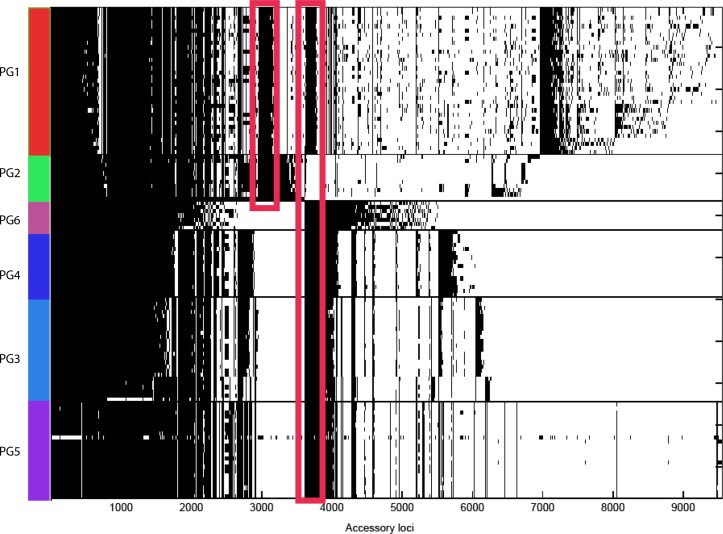
Plot of presence or absence of every genetic locus from the *Y. enterocolitica* pangenome in all sequenced isolates. Genomes are arranged on the *y*-axis according to phylogroup, with individual loci on the *x*-axis. Black indicates presence of the locus in a strain and white indicates absence. The locations of the clusters of loci shared between PG1 and 2, and PG1 and 3–6 are indicated by red boxed areas.

The patterns of acquisitions and deletions, as indicated by the distribution of the accessory genome across the phylogroups, showed distinct patterns of accessory genome maintenance concordant with phylogroup (Fig. 3). Analysis of the data clearly identified two distinct groups of accessory genes: (1) genes shared between PG1 and 2, but absent from PG3–6, and (2) genes shared between PG1 and 3–6, but absent from PG2. The former group includes metabolic genes. PG1 and 3–6 also shared metabolic genes such as ABC transporters, and genes encoding proteins involved in cytochrome *c* and DMSO metabolism. Furthermore, they shared most of the genes located on the ancestral *Yersinia* genus T3SS, *Ygt*, which is found in all species, but has been completely lost in the more acutely pathogenic lineages of *Y. pestis*/*Yersinia pseudotuberculosis* and *Y. enterocolitica* PG2. The pathogenic PG2 and 3–6 exclusively shared surprisingly few chromosomal genes, with a small number encoding regulatory proteins or the nitrate exclusion pathway, in addition to the attachment invasion locus Ail and virulence plasmid pYV. These observations most likely demonstrate key gene loss events occurring in the tree at the ancestral point between PG2 and 3–6. The key gene gain and loss events are indicated on the species phylogeny in Fig. 1. A complete list of phylogroup-specific genes, and genes shared between PG1 and 2, and PG1 and 3–6, is available in Table S2.

### Lowered levels of recombination provide support for ecological separation of the serotype-specific clades

Given the suggestion of reduced gene flow in the low-pathogenic phylogroups, we investigated if levels of homologous recombination differed in and between different phylogroups. We used BratNextGen to detect recombination on core genome alignments (Marttinen *et al.*, 2012). Attempts to detect recombination across the entire dataset proved unsuccessful due to the observed high levels of diversity. Analysis of phylogroup-specific data showed that PG1 and 2 displayed evidence of significant recombination (Fig. S1). However, PG3–6 showed very little detectable recombination within the core genome and events that are detected seemed to be confined to phylogroup-specific admixture events (Fig. S2).

**Fig. 4.**
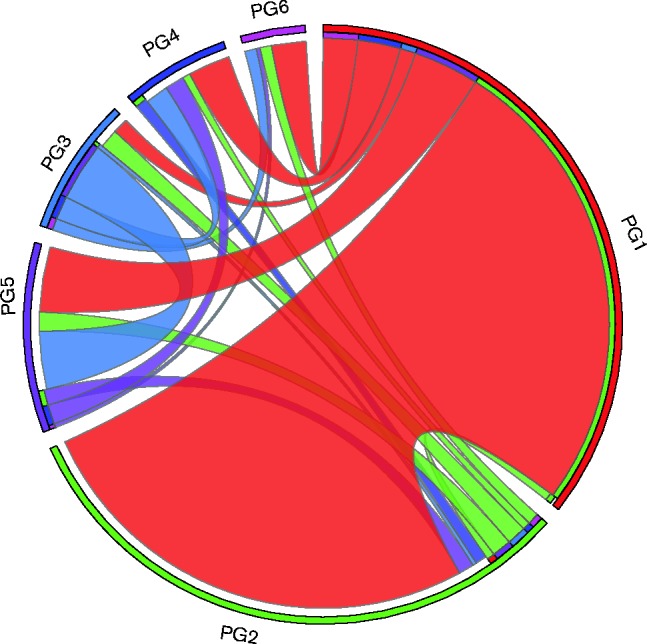
Circos plot showing directional flow of core genome recombination events. For clarity, only inter-phylogroup recombinations are shown due to the extremely large number of intra-PG1 events. Each phylogroup is indicated on the plot. Events donated by a phylogroup are indicated by the colour of the outer circle at each phylogroup segment. Where ribbons touch the outer circles, this indicates a donation; where there is a gap, this indicates receipt.

We inferred the directional flow of the recombination events detected by determining the phylogeny of recombining regions present in the core genome alignment of the entire dataset and plotted the number of events that clearly related a defined donor phylogroup to a different recipient phylogroup. Our analysis excluded within-phylogroup recombination events and events with no phylogenetic signal suggesting introduction from another species (Fig. 4). Our data suggested that PG1 is a reservoir for intra-species homologous recombination in *Y. enterocolitica*, donating genetic material to all phylogroups. Recombination events from PG1 to 2 are most common and our data also showed that intra-species recombination in PG3–6 occurred very rarely. Whilst our method of analysis did not look at each and every recombination block in PG1, the detection of recombining regions flowing from PG2 into PG1, and of a small number from PG3, 4 and 5 into other PG3–6 phylogroups, gives us confidence that our analysis provides an accurate representative picture of the order of gene flow in the species.

## Discussion

Recent work has elucidated the phylogenetic structure of the entire *Yersinia* genus, showing parallel independent evolution towards mammalian pathogenicity in the *Y. enterocolitica* and *Y. pseudotuberculosis* lineages (Reuter *et al.*, 2014). Here, we have further interrogated the genome sequence data of 117 *Y. enterocolitica* isolates to allow us to determine evolutionary events that contribute to the distinct phylogroup structure of the *Y. enterocolitica* species. Our current data provide numerical quantification of the levels of SNP diversity within and between each phylogroup. The levels of SNP diversity within PG3–6 are extremely low, in the range of tens to a few thousands of SNPs. This combined with the tree topology is suggestive of PG3–6 having undergone a recent evolutionary bottleneck (Chain *et al.*, 2004; Croucher *et al.*, 2011; He *et al.*, 2010; Parkhill *et al.*, 2001). Unfortunately, attempts to date any events in the species phylogeny were unsuccessful, with root-to-tip regression analysis suggesting there was no temporal signature remaining in the phylogeny. This was likely to be due to the restricted temporal sampling in the sequenced isolates (Reuter *et al.*, 2014), which although spanning a period from 1974 to 2010 was heavily biased towards strains isolated in the early 2000s. Notwithstanding this, the *Y. enterocolitica* species phylogeny shows striking parallels to the phylogenetic structure expected under the stable ecotype model proposed by Fred Cohan (Koeppel *et al.*, 2008). The stable ecotype model provides an outline of the phylogenetic structure of a population of ecologically separated phylogroups, between which recombination is highly unlikely. The *Y. enterocolitica* phylogeny is akin to a stable ecotype model in which there has been a bottleneck resulting in distinct ecotypes between PG1, 2 and 3–6. It is also suggestive that bottlenecks have occurred in each of PG3, 4, 5 and 6, resulting in six distinct ecotypes within this species.

To fully investigate this notion, we examined the levels of gene loss, gene sharing and recombination in and between the *Y. enterocolitica* phylogroups. Pangenome analyses confirm the previously reported finding that the evolutionary path from PG1 to 3–6 is marked by metabolic reduction (Reuter *et al.*, 2014), with many of the loci identified as unique to PG1 and thought to have been lost by the other phylogroups being involved in intermediary metabolic pathways that use alternative substrates. Phylogroup-specific acquisitions and deletions of accessory virulence factors such as T3SS and secondary flagella clusters were also observed which merit further investigation for their biological role in phylogroup specific phenotypes.

The most phylogenetically concordant signal of gain and loss was seen in the LPS-encoding genes, the clusters for which are phylogroup-specific. Other examples included the virulence factors pYV in PG2–6, and the acquisition of the high-pathogenicity island and an additional T3SS by all members of PG2 (Reuter *et al.*, 2014). Most of the phylogroup-defining regions of difference are the result of gradual decay of loci that are presumably no longer under any form of selective pressure to be maintained in the population. The most likely explanation for this is because the phylogroups are being exposed to different environmental selective forces. Such a phenomenon is widespread in bacteria where gene loss occurs as a response to a shift in ecology, and is best exemplified in the evolution of *Salmonella* Typhi, *Salmonella* Gallinarum, *Mycobacterium leprae* and *Y. pestis* (Chain *et al.*, 2006; Cole *et al.*, 2001; Holt *et al.*, 2008; Langridge *et al.*, 2015).

The pangenome analysis suggests that PG1 has an open genome and frequent flux at the accessory gene level (Rasko *et al.*, 2008); this is also largely true of PG2. However, the pangenomes for the other ‘low-pathogenic’ phylogroups, PG3–6, were essentially closed, suggesting that these low-diversity lineages are not accessing the considerable accessory gene pool available to other members of this species. Core genome recombination levels mirrored this observation, with PG1 undergoing extensive core genome recombination both within and between phylogroups, with a slight decrease in the PG2 lineage and then very low levels of detectable recombination in PG3–6. This observation is consistent with recently emerged, niche-restricted pathogenic lineages (McNally *et al.*, 2013b; Willems *et al.*, 2012). Directional analysis of the recombination shows that PG1 acts as a reservoir for core genome recombination and that the low-pathogenic phylogroups act as infrequent acceptors of genetic exchange, but extremely rarely as donors. Such a pattern might be expected if the genomic dataset was biased towards PG1 isolates; however, our dataset comprises just 26 % PG1 genomes, strengthening the suggestion that PG1 acts as a frequent donor for recombination to the rest of the species. However, it may be the case that PG1 dominates as a recombination donor due to having a much larger population size compared with the other phylogroups. It is well known that PG1 is ubiquitous and the most commonly isolated *Y. enterocolitica* from non-human environments (McNally *et al.*, 2004), whilst PG3–5 are the most commonly isolated from human infections, with PG2 rarely isolated and PG6 niche-restricted (McNally *et al.*, 2004). Therefore, it is possible that our recombination observations are a reflection of the population sizes of the different *Y. enterocolitica* phylogroups.

All phylogroups of *Y. enterocolitica* can be isolated from the intestinal tracts of cattle, sheep and pigs, as well as porcine tonsil tissue (with the clear exception of PG6, which so far has only been seen in hares). Moreover, PG2–5 are isolated from human disease cases showing identical pathology and symptom severity, despite the differential levels of virulence in the mouse infection model. Given the common reporting of *Y. enterocolitica*, it would be reasonable to expect that chance encounter between different phylogroups in the same habitat would be common and therefore opportunities for recombination to occur would be frequent. However, our evidence suggests that recombination does not occur frequently between phylogroups, and that when it does, it is primarily unidirectional with PG1 donating genetic material to PG2–6.

The data presented here suggest that the distinct phylogroups of *Y. enterocolitica* may be ecologically separated. This draws parallels with a number of published examples of bacterial ecotype evolution and separation, including the phylogenetic structure expected under the stable ecoptype model (Koeppel *et al.*, 2008). A study of *Escherichia coli* isolated from environmental reservoirs showed that when compared with human and animal isolates, core genome recombination was only observed between environmental isolates or between human and animal isolates, but never between environmental and human/animal isolates (Luo *et al.*, 2011). This was highly suggestive of ecological barriers to gene flow in *E. coli*, an observation further suggested by analysis performed by our group on *E. coli* ST131 and the emergence of a multidrug-resistant clone (McNally *et al.*, 2013b). Our study also draws parallels with observations made in host-restricted lineages of *Campylobacter jejuni* where ecological barriers restrict recombination and gene flow between ecotypes (Sheppard *et al.*, 2014). Our data and those discussed above fit with the idea of the ‘reverse ecology’ approach hypothesized to predict distinct ecological units in bacterial species from genomic and phylogenetic data (Shapiro & Polz, 2014). Using this approach, distinct ecotypes have been identified within the species *Vibrio cyclitrophicus*, each exhibiting ecotype-specific islands of SNP diversity (Shapiro *et al.*, 2012). The approach also identified distinct genetic content in *Prochlorococcus* isolated from Atlantic and Pacific waters, resulting in distinct ecotypes (Coleman & Chisholm, 2010).

In summary, it appears that there are deep divisions between the *Y. enterocolitica* phylogroups that manifest in high core genome diversity and distinct lineage-defining LPS operons and O-antigens. There are clear barriers to genetic exchange with the ubiquitous PG1 acting as a recombination donor, albeit infrequent, for the other phylogroups and yet there is rare exchange between the other phylogroups. The genome analysis has also revealed molecular markers that suggest that some lineages have undergone reductive evolution. Together with the topology of the species tree, this suggests that the *Y. enterocolitica* lineages PG2–6 have specialized ecologically and in the process show signs of having gone through a selective sweep. It was not possible to determine when this occurred. However, what is clear is that our data raises questions about the accepted dogma of *Y. enterocolitica* ecology and that more exacting ecological studies may be of merit to better understand this important group of pathogenic bacteria.

## Supplementary Data

285 Supplementary Data

## Supplementary Data

108 Supplementary Data

## Supplementary Data

56 Supplementary Data
