# Supplementary figures and images for: Directional gene flow and ecological separation in Yersinia enterocolitica
Source: Microb Genom. 2015 Sep 29;1(3):e000030. doi: 10.1099/mgen.0.000030 (PMC5320568; doi:10.1099/mgen.0.000030)

**PG1**

**PG2**

$r/m = 7.43$

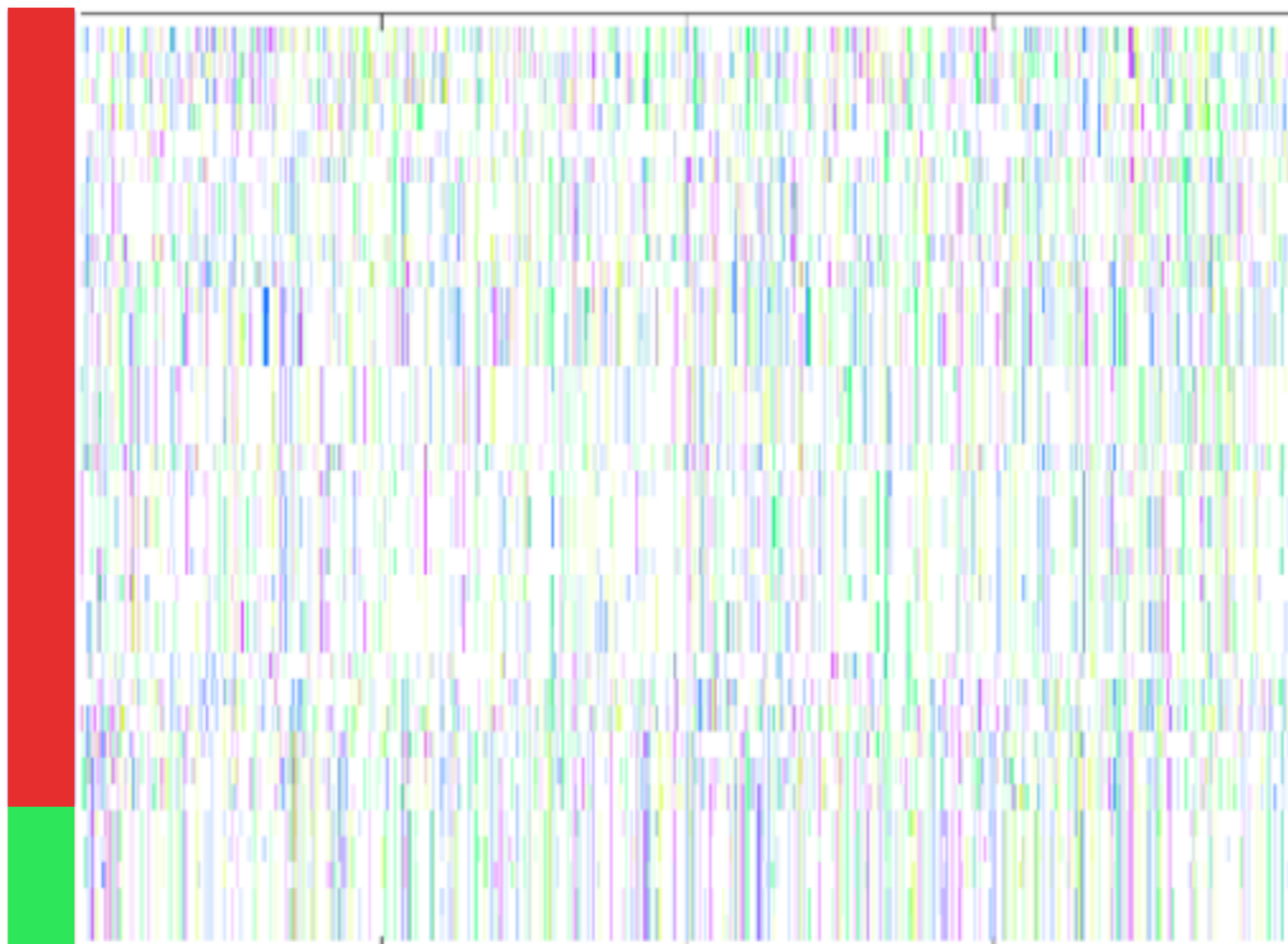

Supplement: Supplementary file 1 — Supplementary Data [file mgen-01-30-s001.pdf]

r/m = 0.53

**PG4**

**PG3**

**PG6**

**PG5**

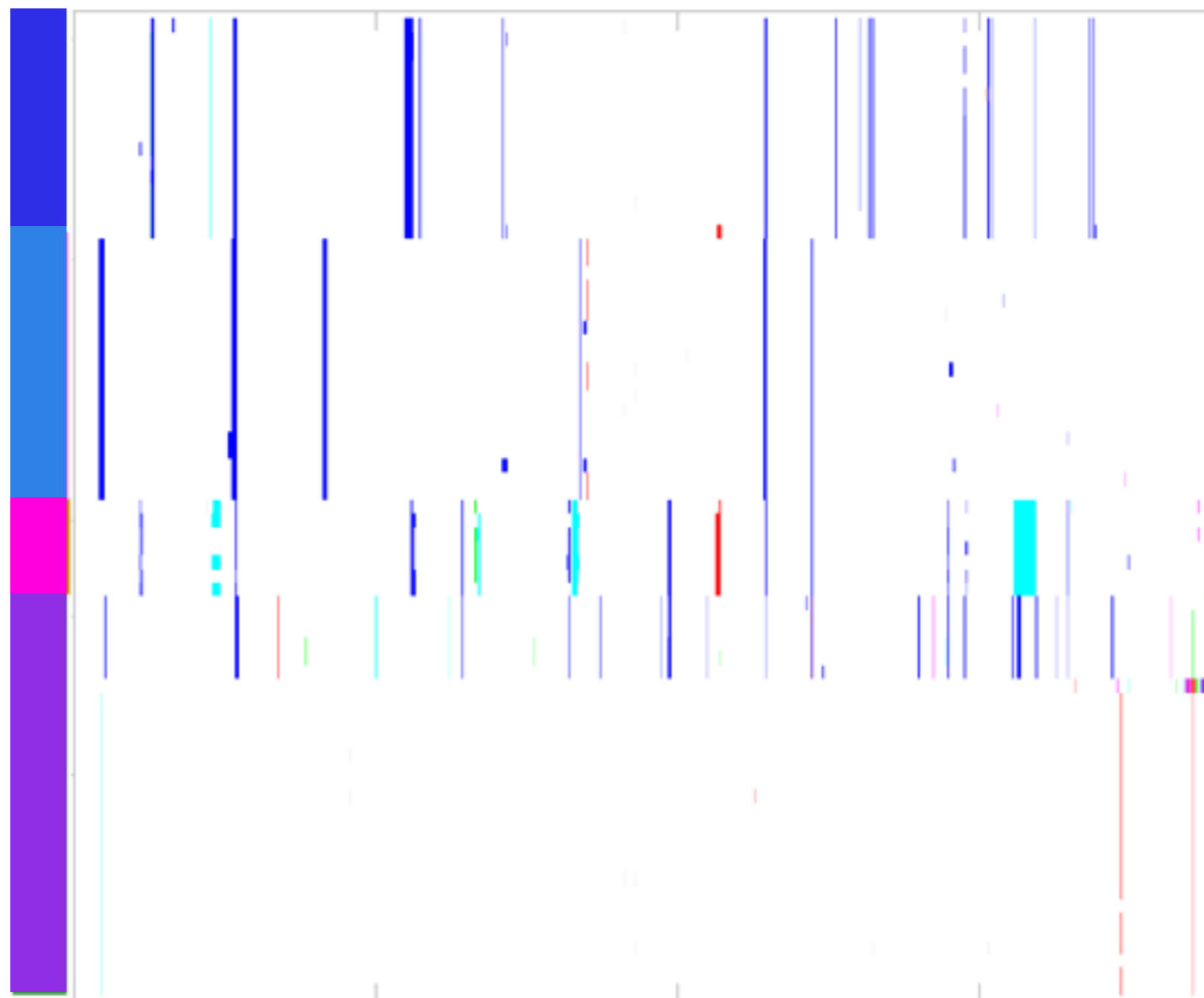

Supplement: Supplementary file 2 — Supplementary Data [file mgen-01-30-s002.pdf]
